# Quasi-Linear Vacancy Dynamics Modeling and Circuit Analysis of the Bipolar Memristor

**DOI:** 10.1371/journal.pone.0111607

**Published:** 2014-11-12

**Authors:** Isaac Abraham

**Affiliations:** Cloud Platform Group, Intel Corporation, Dupont, Washington, United States of America; and Department of Electrical Engineering, University of Washington, Seattle, Washington, United States of America; Institute for Materials Science, Germany

## Abstract

The quasi-linear transport equation is investigated for modeling the bipolar memory resistor. The solution accommodates vacancy and circuit level perspectives on memristance. For the first time in literature the component resistors that constitute the contemporary dual variable resistor circuit model are quantified using vacancy parameters and derived from a governing partial differential equation. The model describes known memristor dynamics even as it generates new insight about vacancy migration, bottlenecks to switching speed and elucidates subtle relationships between switching resistance range and device parameters. The model is shown to comply with Chua's generalized equations for the memristor. Independent experimental results are used throughout, to validate the insights obtained from the model. The paper concludes by implementing a memristor-capacitor filter and compares its performance to a reference resistor-capacitor filter to demonstrate that the model is usable for practical circuit analysis.

## Introduction

Memristive dynamics in chemicals has been reported in experimental literature from at least the late 1960s in conjunction with the study of thin films [1]. The mathematics behind the memristor was presented by Chua in 1971 [2]. It was manufactured by Hewlett-Packard's (HP) Williams et al. in 2008 accompanied by two important papers that are ubiquitously referenced [3], [4]. The name memristor is an abbreviation for memory resistor. It is a two-terminal resistor that retains memory of the last known resistance prior to removal of the programming stimulus. Physically the device consists of two metal end plates with a chemical sandwich that has mobile vacancies also referred to as defects. For example a common chemical species is titanium dioxide where some of the compound molecules might lose a positive oxygen ion resulting in negatively charged 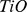 which can be thought of as forming the defect structure [3], [4]. Gathering the vacancies to any one end plate results in a high resistance and distributing them makes the device exhibit low resistance [3]. Choosing the right chemical species to provide the mobile vacancies makes it possible to integrate the device into a CMOS substrate. In general, and without considerations of any high electric field induced breakdown, the memristor would seem very amenable to device length scaling. The possibility of integration with CMOS technologies combined with the dimension scalability make them good candidates for use as high-density memory elements, where the low and high resistance states can represent binary data.

Identifying the performance limiting parameters is an important outcome for any modeling effort. Clearly specified variables, computability and clarity are essential for a usable model. Portability to a Simulation Program with Integrated Circuit Emphasis (SPICE) environment will allow specialist circuit designers to investigate circuit networks that incorporate memristors.

The scope of memristor literature has expanded remarkably following the fabrication work by Williams [4]. Papers on the topic may be broadly classified as modeling or circuit implementations that derive from Strukov et al.'s dual variable resistor model [3]. Within the modeling and circuit category, one will find theoretical and experimental papers. Strukov and Williams' are the two contemporary papers that introduce the idea of the memristor being composed of dual variable resistors with a low and high resistance. These two papers also form the fountainhead for numerous contributions by various authors.

Joglekar and Wolf [5] mathematically address the moving boundary between the low and high resistance regions, device resistance and non-linear dopant drift toward end plates. Non-linear drift is tackled using window functions that have a tuning knob (variable) to help adjust the vacancy velocity toward the end plates. Their paper has some explorations of circuits using memristors.

Biolek et al. [6] take the basic model from [3], discuss the implications of nonlinear dopant drift from [5], and suggest enhancements to the window function. They also present a compact SPICE model.

Kim et al. [7] present a memristor emulator circuit built using discrete components. The paper is based on Chua's work on the memristor and has an extensive repertoire of I–V curves generated with the emulator.

Waser et al. [8] methodically classify various physical phenomena that could result in memristive behavior. From the discussion therein, it is clear that bipolar memristance resulting from vacancy dynamics is a subset of the broader phenomena of memristance. The paper also serves as a survey of experimental results, illustrated with Scanning Electron Microscope (SEM) captures and containing chemistry intensive discussions. The authors recognize the development of a large body of literature where the transport of ions is considered essential for resistive switching. This transport results in the depletion or enrichment of vacancies in a given volume of the memristor, resulting in a considerable change in electronic conductivity.

Meuffels and Soni [9] present a strong case for why the memory resistor model as presented in [3] is unrealizable as a practical device. Their work points out that to arrive at the equations in [3], the concentration in the on and off regions of the memristor must remain unchanged despite the moving boundary. Such an (enforced, impractical) assumption will lead to the equations in [3] and also to an undesirable, unstable physical condition at the boundary between the on and off regions. These criticisms are readily redressed by this manuscript, in the last paragraph of this section.

Strachan et al. [10] present a study on tantalum oxide memristors. They determined that the on state was metallic while the off state was best described by the Frankel-Poole relationship. The authors model the device conductance as a parallel combination of the two phases. Strachan's work may be more suited as a demonstration of a phase change, unipolar memristor, while this work seeks to address only bipolar memristance deriving from memristive dynamics.

Nardi et al. [11] take a unique approach with the vacancies subjected to the continuity equation accompanied by a numerical solution. The two-part paper has experimental results in Part-I, which are then compared to the results of the numerical model from Part-II, with some success over the current-voltage (I–V) curves under consideration. Nardi et al. [11] may be one of very few papers that have attempted a modeling technique that reaches into the vacancy levels, rather than start with the dual variable resistor abstraction.

This paper recognizes that many physical phenomena such as electrochemical, defect or vacancy migration, stoichiometry and phase change can exhibit memory resistance [8]. The modeling presented here is confined to bipolar memristance arising from vacancy migration. This work avoids the unstable physical boundary that may result from the modeling in [3] (as pointed out by Meuffels) by proposing a refined definition for the boundary that separates the device volume with more vacancies from the device volume with fewer vacancies. This model allows the vacancy concentration to vary non-linearly between the end plates. The vacancies are also recognized as being able to diffuse and lose their stored state over a prolonged period of storage with no applied voltage. The volatility will be driven by the much smaller diffusion constant of the vacancies compared to the mobility when vacancies drift in the presence of an applied electric field. At a high level of abstraction, this paper is similar to Nardi et al. [11] in seeking a solution that is independent of the dual variable resistor model, yet transcends the vacancy and circuit levels of abstraction. This paper is different from [11] in that the proposed model in the form of a single partial differential equation (PDE) is solved analytically, to yield computable equations for vacancy and circuit dynamics. The generally accepted idea of nonlinear [3] vacancy transport [8] between end plates suggests that some form of the non-linear transport equation should be able to model the memristor phenomenon. The literature survey reveals a variety of equations that quantify different pieces of the memristive phenomena but not a unifying model. The goal for this paper is to present a single governing equation accompanied by an analytical solution that can be manipulated to yield known memristive characteristics including the ubiquitously referenced dual variable resistor circuit abstraction.

## Methods

### A. Assumptions

The following assumptions provide a framework for how vacancies evolve with time, inside the memristor. Nardi et al.'s [11] experimental work shows that resistance did not depend significantly on the device area. Since the top-plate may contact the ambient, this data is taken to indicate that there is minimal or zero ingress/egress of vacancies from within the device boundaries [8]. Taking into consideration, the “bubbles in glass” analogy from [4] and the fairly repeatable empirical I–V data from many sources it may be assumed that vacancies are drifting and accumulating toward the attracting end plate, with an accumulation boundary that separates the region with lots of vacancies from the region without many vacancies. The assumptions and related references are concisely stated as follows,

- Vacancies are conserved [8], [11].
- Vacancies accumulate to one end plate to a maximum normalized concentration of unity and vacancies dissipate from the opposite region to a minimum normalized concentration of zero [3], [4].
- A non-linear drift mechanism governs the movement of these vacancies between end plates [5], [6], [9]. The author recognizes that nonlinear drift will be modulated by diffusion and thermal effects to some extent as mentioned by Strachan et al. For the first steps with this derivation, drift is considered dominant when vacancies move toward the one or the other end plate. This should not dilute the essence of the phenomenon because the vacancies conclusively drift in the presence of the applied electric field [3].

The derivation uses normalized vacancy concentration to remove ambiguity relating to the value of mobility, precision of the programming voltage, the total quantity of vacancies in the material etc. associated with disparate sources. A subset of data is presented with the normalization removed, to compare to published results [11], [12], [13] and show that the normalization is not hiding any undesirable side-effects.

### B. The Continuity Equation and Solution

Having identified the common thread of drift and conservation from literature, this paper proposes the variable co-efficient advection or transport equation as the governing PDE.
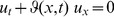
(1)


In (1), 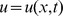 and is the normalized vacancy concentration at a point inside the memristor. The coefficient of 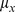 is the variable velocity 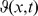 which will accommodate the “non-linear dopant drift” that is anticipated by the many references [5], [6], [9]. A simpler equation 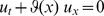 is a teaching aid and has the solution 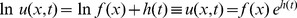. Additionally the author observes that a simple PDE such as 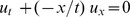 which accepts 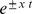 also accepts 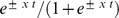 as a solution. The second form of the solution (with the positive exponents) has the desirable property that it asymptotically approaches zero for 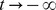 and unity as 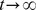 forming the basis for the proposed solution to the evolution of (normalized) vacancy concentration within the memristive device. With the aforementioned guidance, a test solution of the form 
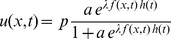
 is proposed for the PDE (1), such that at 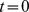, the concentration is some constant representing the equally distributed value. The variable 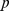 is an additional knob that limits the maximum concentration to less than unity due to forces of repulsion [14]. Except where explicitly mentioned, *p = 1*.

**Figure 1:**
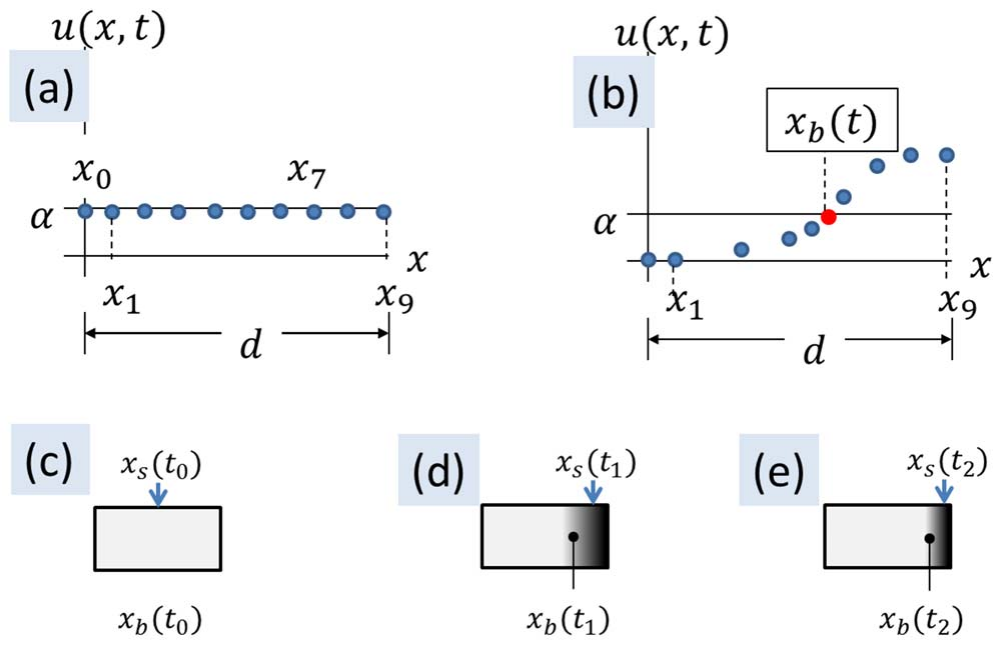
The vacancy accumulation and symmetry boundary. (a) The accumulation boundary is non-existent when the vacancies are distributed through the volume of the memristor. (b) The boundary 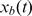 becomes well defined when the vacancies accumulate to one end plate. (c–e) Sketch of the accumulation boundary and vacancy symmetry boundary evolving as the vacancies accumulate to one end plate.



The concept of accumulation boundary is read in [4], [5]. With reference to Fig. 1(a), the author observes that when the vacancies are evenly distributed, it is impossible to distinguish any point along the device from any other, from the point of view of concentration. As the vacancies accumulate to one end plate, there will always be a point 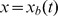 which has the same normalized concentration as the initial distributed value 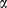. It is assumed that there is no discontinuous jump from zero to unity at the accumulation boundary, at any time. This location 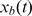 is the refined definition of the accumulation boundary. Since the numerator and denominator must be dimensionless, consider just the numerator when attempting to expand the various functions in 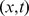. Taking the natural logarithm results in 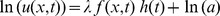, where the term 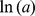 can be evaluated after applying the initial conditions at 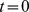. The first term can evaluate to a normalized concentration like the other terms only if 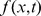 represents a velocity, 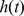 represents time and 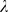 is the normalization factor. With this insight, assign 
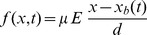
, where 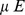 is the velocity of vacancies when far away from the influence of the device boundaries or the accumulation boundary. The term 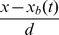 modulates the free-space velocity with the normalized distance of a point from the accumulation boundary. The assignment of 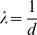 and 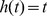 makes the exponent dimensionless. The true vacancy velocity at any location will be given by the velocity term 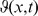 in the governing equation (1). The complete solution is presented as follows with *p = 1*.

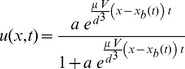
(2)where,


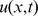
 is the normalized concentration at some 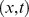 (unit less),


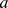
 is a yet-to-be determined true constant coefficient (unit less),


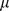
 is the vacancy mobility 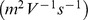,


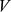
 is the programming DC voltage (V),


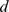
 is the device length 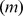,


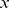
 is the length along the device,


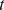
 is time and


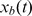
 is the accumulation boundary.

Given that the memory resistor is a flux controlled device, where flux is the time-integral of the voltage, the solution can be rephrased in terms of flux with quantities in 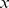 simultaneously normalized as,
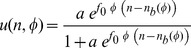
(3)where,


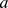
 is the constant coefficient,


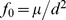
 is a natural frequency,


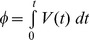
 is the flux and


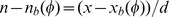
, is the normalized distance of a point inside the device, from the flux dependent accumulation boundary.

The equations as presented simplify the concepts while being scalable to accommodate temperature effects. Mobility can be expanded using the Einstein-Smoluchowski relation which relates diffusion constant 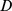 to the electrical mobility 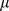 of particles as 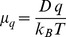, where 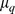 is the mobility of the charged particle (vacancy), 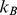 is the Boltzmann constant, 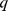 is the electrical charge of the particle (vacancy) and 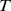 is the temperature. In summary, the derivation of the solution is based on the author's observations about the PDE and material from [15].

### C. Solution Verification

#### 1. Initial Condition

The initial condition can be tested by substituting 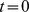 in (2).
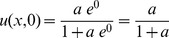



The above can be used to calculate 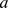, by equating it to the distributed initial (normalized) concentration 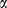. Upon solving, the solution is 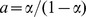.

#### 2. Final Condition

The final condition has two parts to be evaluated, one at each end of the device. The following calculation assumes that vacancies are drifting and accumulating to the right side of the device.
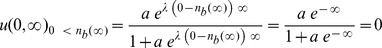





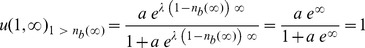



The two normalized conditions are satisfied.

## Results

This section derives and provides visualization for various dynamics of interest such as the vacancy accumulation boundary, vacancy symmetry boundary, vacancy velocity and device resistance. In the accompanying figures, the plots were generated with an excitation waveform described by 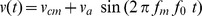. The common-mode of the wave is 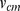 and the amplitude is 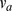. Variable 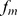 denotes a multiple of the natural frequency of the memristor 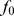 as defined in (3). The parameter values used for all calculations and plots are in Table 1. When a plot or calculation uses different values other than from the table, the reason and the specific value used are called out.

**Table 1:** List of parameter values used for calculations.

#	Symbol	Parameter	Units	Value
1	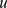	Normalized vacancy density	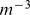	-
2	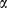	Normalized average concentration	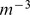	0.2
3	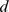	Device length	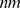	32
4	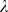	Inverse device length	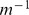	-
5	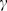	Resistance of pristine chemical sandwich species	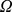	100
6	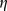	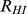 limiter (optional computational aid)	-	0.0
7	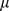	Vacancy mobility	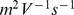	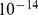
8	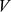	Programming voltage	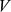	1
9	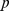	Packing factor	-	1
10	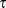	Transition time	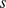	-

### A. Vacancy Velocity

For a constant programming voltage 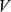 and constant mobility 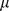, the proposed solution (2) satisfies the PDE (1) when, 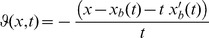
(4)


**Figure 2:**
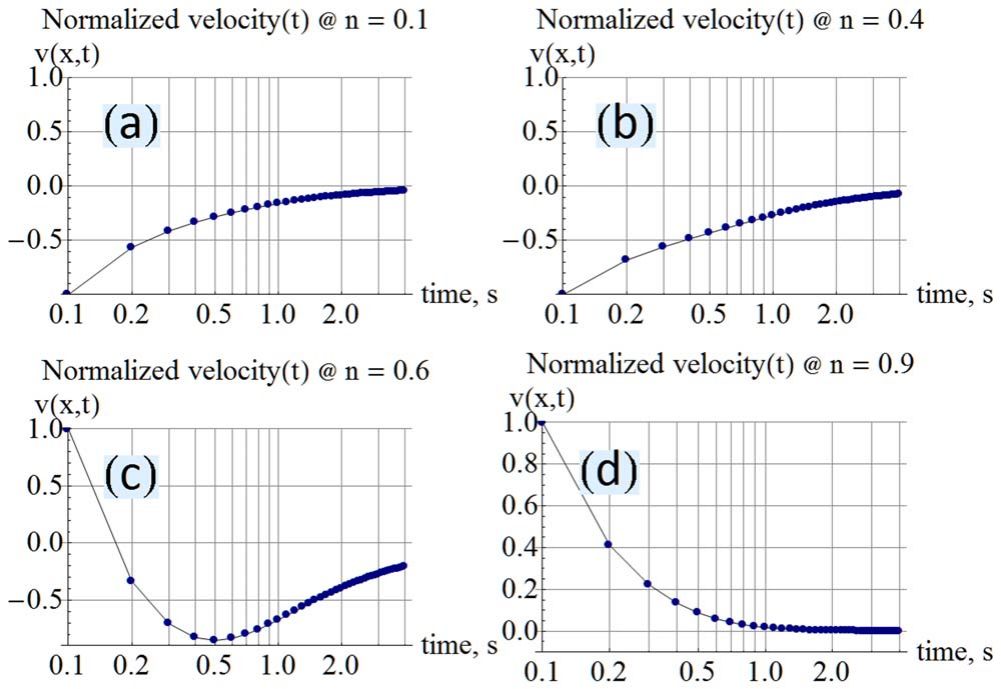
Vacancy velocity within the memristor. (a) Vacancy velocity is negative close to the left end plate at 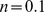 signifying evacuation of vacancies. (b) Even at 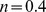, the vacancies are evacuating the location because the device was originally initialized to a low resistance and the accumulation boundary materializes past the 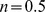 position. (c) Shows an intermediate location 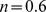 which initially experiences an inrush of vacancies (with positive velocity) and then loses vacancies (with negative velocity) as the accumulation boundary transits the location. (d) A location very close to the right end plate always accumulates vacancies. The velocity in all panels asymptotically approach zero with time.

This method of solving for 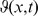 produces a computable formula for velocity since it is not possible to enforce the velocity of vacancies inside the device. Fig. 2 plots (4) at various locations, for 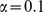. Negative velocity indicates dissipation of vacancies while positive velocity indicates accumulation. Fig. 2(a) and Fig. 2(d) show dissipation and accumulation at very close to the left and right end plates respectively. Fig. 2(b) shows dissipating vacancies because the device has been initialized to its lowest resistance state with the accumulation boundary at infinity as in Fig. 1(c) and the only possible outcome when vacancies drift to the right, is for the location *n = 0.4* to lose vacancies. In Fig. 2(c) the normalized location *n = 0.6* exhibits an initial gain of vacancies with positive velocity when vacancies from the left side rush in. As the accumulation boundary transits through this point, the velocity becomes negative (representing outflow), reaches a negative maximum, and asymptotically tends to zero as time increases.

### B. Accumulation Boundary

The formula for accumulation boundary is derived by integrating *p u(x,t)* over the volume of the device, equating to the estimated total vacancy count in the device and solving for *x_b_(t)* (or the normalized 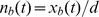). The result is shown here with 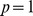 for simplicity where 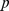 is the additional knob that limits the maximum concentration to less than unity due to forces of repulsion [14]. 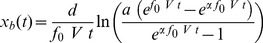
(5)


**Figure 3:**
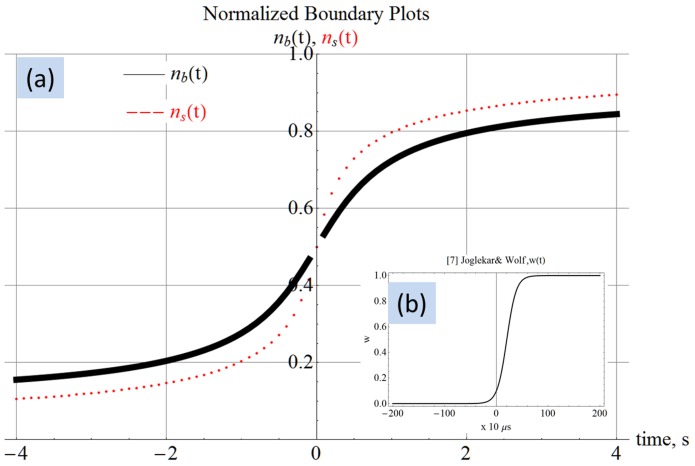
Computed normalized accumulation and symmetry plots. The normalized plots of the vacancy accumulation boundary and symmetry boundary show that while the accumulation boundary “tends to infinity” when the vacancies are distributed (at 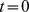), the symmetry boundary is always within the physical ends of the device.

This derivation assumes that the total number of vacancies is known from the stoichiometry of the chemical species. The accumulation boundary is mentioned with a similar equation in [5]. The bold thick plot in Fig. 3(a) shows the simulation for the normalized accumulation boundary. The missing segment at 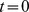 is because the boundary has traveled to infinity according to the refined definition, illustrated in Fig. 1(c). Fig. 3(b) shows a plot of the boundary from [5]. There is a simple time shift between Fig. 3(a) and Fig. 3(b) because of how the models are initialized. The timescale is different because Joglekar's boundary is shown for a 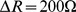, while the calculation in this paper is for a 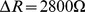 and involving an assumed initial vacancy concentration of 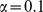. Due to the unknown parameters, the only intent here is to show the agreement in the general shape.

### C. Vacancy Symmetry Boundary

This paper introduces a new concept of vacancy symmetry boundary to offset the inconvenience of having the accumulation boundary tend to infinity when the vacancies are evenly distributed as sketched in Fig. 1(c). The vacancy symmetry boundary 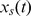 is a location that has an equal number of vacancies to either side of it. The formula is derived similar to 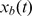 with 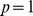 and 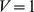.
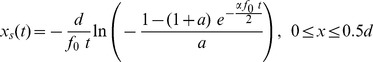
(6)The vacancy symmetry boundary is shown sketched in Fig. 1(c–e) and marked with the blue arrow. Fig. 3(a) plots a simulation of the vacancy symmetry boundary with a red dotted line. Notably the vacancy symmetry boundary always evaluates to a position within the boundary of the device, unlike the accumulation boundary which tends to infinity when the vacancies are evenly distributed. The infinity signifies that there is no boundary in the traditional sense because the entire volume of the device has the same vacancy concentration.

### D. Resistance

The model in this paper stands apart from other contributions in that it derives a resistance equation, rather than starting out from the dual variable resistor model with known 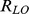 and 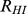. The derived resistance equations reveal dual variable resistors and some additional interesting characteristics.

For the derivation, the chemical species with the mobile vacancies is considered to be like an electrolyte. The similarity comes from the fact that a vacancy concentration *u* 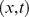 at a location translates into resistance just like in an electrolyte. With reference to sketches from [4] and empirical data in general, a low resistance is associated with vacancies being distributed through the length of the device. In keeping with these observations, the resistance equation at any location inside the device boundaries is proposed to be,
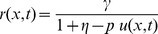
(7)


In (7) 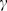 is the resistance in a region that is devoid of any defects. The variable *η* is an arbitrary computational knob to limit the maximum resistance and prevent a singularity if 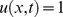. The value of 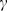 may be known from empirical data or the chemical composition of a pristine device with no vacancies.

**Figure 4:**
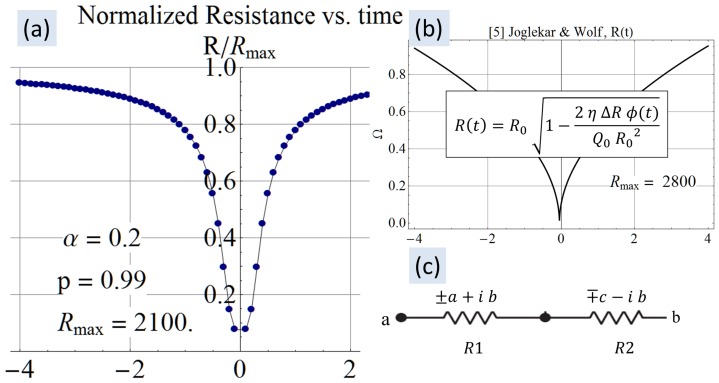
Computed resistance-time plot. Panel (a) shows the resistance-time plot generated using (8) in agreement with panel (b) showing similar results from [5]. The complex 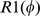 and 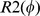 in panel (c) is a manifestation of phase information as observed by Chua [16].



**Figure 5:**
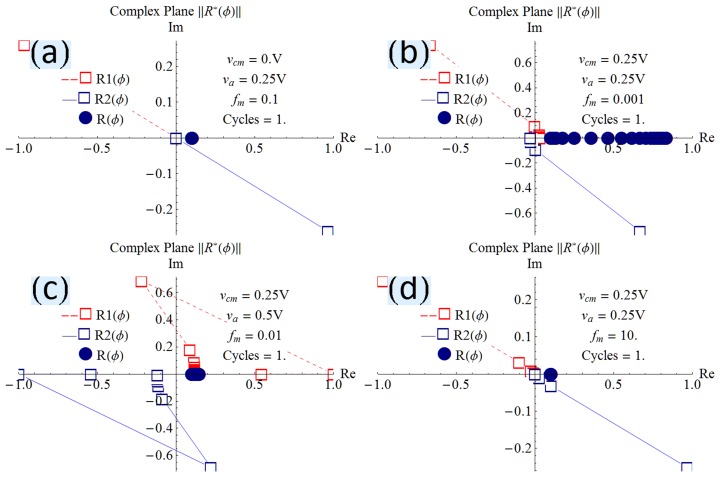
Computed dual variable resistors. The resistors 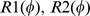 and 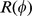 in the complex plane show that the composite resistance 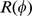 is always real and positive.

The resistance across the device is determined by integrating (7) over the device length, resulting in 
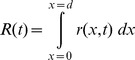
. The normalized resistance-time plot in Fig. 4(a) shows two resistance states. The plot in Fig. 4(b) was generated by the author using the equation from [5] shown along with the plot. The similarity confirms that the proposed model is able to reproduce the transient behavior from published works. The plot uses 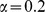 from Table 1 to facilitate a closer approximation to [5]. The maximum value and the timescale (in seconds) in which this transition happens are reasonably close to that from [5]. Rearranging terms after integration produces two constituent resistors similar to the dual variable resistor model.

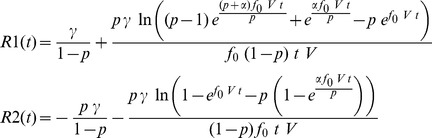
(8)All computed results are generated with 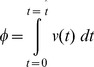. In the special case where voltage is DC 1 V, then it is possible to write 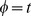. The following discussion may use 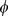 and 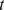 interchangeably. The two resistors in (8) individually evaluate to complex quantities that may be represented as 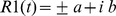 and 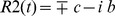 as in Fig. 4(c). In this complex representation the real parts always evaluate such that the positive real part (either 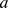 or 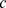) is always greater than the negative real part. Therefore, when these resistors appear in series in the dual variable resistor model, the result is a positive composite resistance for all 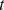. The resistors 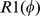 and 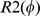 are complex because they individually contain phase information as observed by Chua [16]. Fig. 5 shows the normalized resistances in the complex plane. Fig. 5(a) shows that when forced with a programming sinusoid having a common-mode 0 V and one-tenth the natural frequency of the device, the constituent resistors 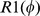 and 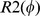 have the form 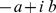 and 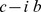 respectively. The filled navy blue circle represents the composite normalized resistance 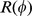, which is shown to be at about one-tenth the maximum possible value. Fig. 5(b) demonstrates that with a programming common-mode and amplitude of 0.25 V, at a frequency of one-thousandth the natural frequency, the constituent resistors 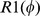 and 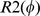 evolve significantly. 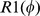 transitions from 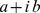 to 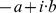, where the real part has changed from a positive resistance to a negative resistance. 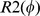 transitions from *-a-i b* to 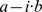, where the real part has changed from a negative resistance to a positive resistance. The composite resistance has a wider spread because the resistance is evolving quickly even in the one cycle under consideration due to the low frequency. Fig. 5(c) is different in that the amplitude of the sinusoid is larger than the common-mode offset, causing negative voltage excursions which delay the accumulation of flux. Consequently, the composite resistor has not evolved much, as evidenced by the small range of values occupied by the filled navy blue dots on the positive real axis. Fig. 5(d) shows the resistors when subjected to a programming voltage at ten-times the natural frequency. The constituent resistors trace a straight line and the composite 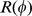 has not evolved at all. This is the expected behavior because the vacancies in a memristor subjected to a high frequency will not have enough time to dislodge from their original positions in any of the waveform cycles [5]. All panels of Fig. 5 conclusively show that the composite resistance is always real and positive.

**Figure 6:**
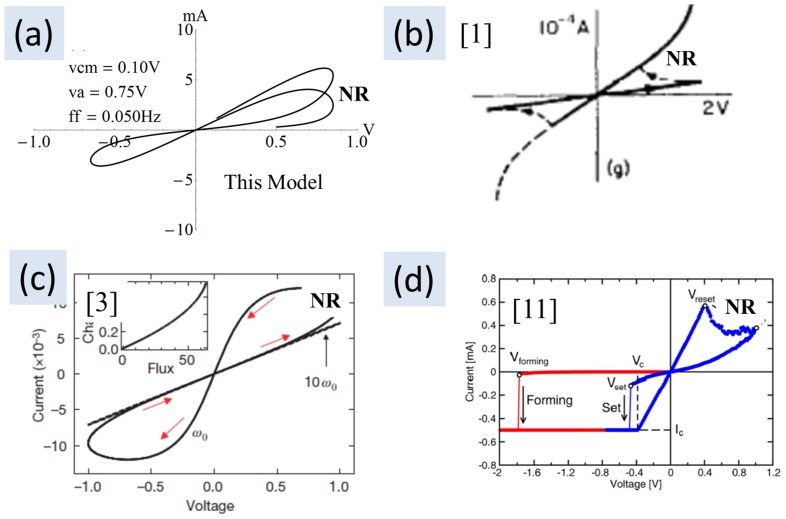
Negative resistance. Experimental and simulated I–V curves marked up “NR” showing the negative resistance region. This negative resistance is not useful in designing an oscillator because of the brevity of existence.

The excursion of the real part of the constituent resistors between positive and negative numbers is important. It explains why there is seemingly a negative resistance region in the I–V curve for the memristor. Since 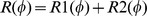, the sum will exhibit an increase or decrease depending on the rate at which 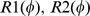 are evolving due to phase differences between these resistors. The semblance of negative resistance lasts only for a brief range of the sweep voltage, and may be practically unusable for building an oscillator as observed by Chua [16]. The primary objective in this subsection is to emphasize that the independent methods presented here are able to mathematically show the underlying reason for the existence of a negative resistance region in the memristor I–V curve. Fig. 6 shows various experimental and simulated I–V curves with their negative resistance region marked up as “NR”. Fig. 6(a) shows an I–V curve produced using (8). The plot traces an asymmetric first cycle because of the common-mode offset of 0.1 V. The second lobe in quadrant 1 has lower current caused by the increasing resistance. The plot was abruptly stopped before reaching the origin to clearly show the (higher resistance) direction in which the trace was progressing. Fig. 6(b) shows an experimental I–V curve from Argall [1]. Fig. 6(c) shows an I–V curve from Strukov [3] and Fig. 6(d) shows an experimental I–V curve from Nardi et al. [11]. Fig. 6(a) should also serve to demonstrate that the model proposed in this submission can produce the ubiquitous bow-tie I–V curve that appears in every memristor paper.

**Figure 7:**
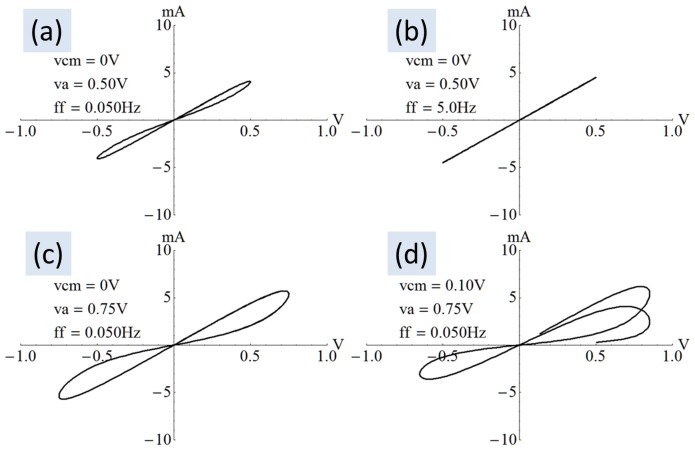
Current-voltage curves. A collection of I–V curves generated using (8) where the device was initialized into the low-resistance state. (a) A reference curve with zero common mode. (b) At the natural frequency the I–V curve becomes a straight line like a simple resistor. (c) With a large amplitude, the lobes take on odd shapes. (d) The lobes are offset and asymmetric when the programming voltage has a common-mode offset. The curve is clearly seen to transition from low to high resistance.

Fig. 7 shows a variety of I–V curves produced using (8) and varying the common mode (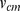), amplitude (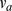) and frequency (*ff  =  f_0_ f_m_*) of the stimulus. The natural frequency of the device 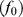 was about 5 Hz. Fig. 7(a) shows symmetric lobes and may be considered as a reference for the discussion. Fig. 7(b) shows the impact of increasing the frequency to approximately the natural frequency of the device, whereby the I–V curve became a straight line akin to a traditional resistor. In Fig. 7(c), the amplitude of excitation was increased while keeping the frequency at one-hundredth the natural frequency resulting in deformation of the lobes compared to Fig. 7(a) and also a higher resistance for the same applied voltage. For example, the lower trace of the lobe in quadrant 1 of Fig. 7(a) shows about 3.5 mA at 0.5 V (equivalent to 143Ω), whereas Fig. 7(c) presents about 2 mA at 0.5 V (equivalent to 250Ω). Fig. 7(d) shows multiple sweeps progressively accumulating higher resistance.

### E. Switching Time

Switching or transition time is the time it takes for a memristive device to transition from low resistance to high resistance or vice versa. This author designates the ratio of high to low resistance as 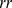 for resistance ratio. Algebraic manipulation of (7) with 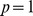 and 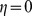 yields 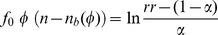, where 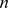 is the normalized distance and 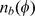 is the normalized accumulation boundary. Substituting 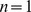, 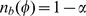 and 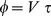, the equation for switching time becomes,
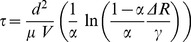
(9)


The term 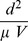 is already seen in [14], [17] and is referred to as the primary formula in the following discussion. The term 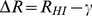 represents the difference between the highest resistance achieved by the memristor when all vacancies are pulled to one end plate and 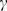, the resistance of a device with no vacancies in it. For practical purposes 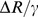 is a resistance ratio. The modulating term 
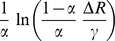
 is now investigated. When there are no vacancies in the device, one expects that it will take an infinite time to switch to any resistance ratio. 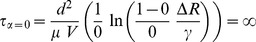



**Figure 8:**
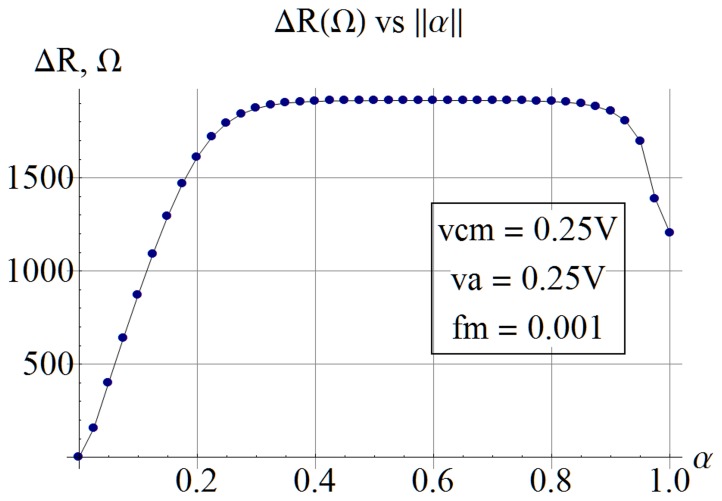
Vacancy concentration dependence of switching resistance. Resistance switching range decreases for very low and high values of vacancy concentration. Therefore excessively increasing the concentration of vacancies is not a viable option for decreasing switching time if the switching range is not expected to decrease.

**Figure 9:**
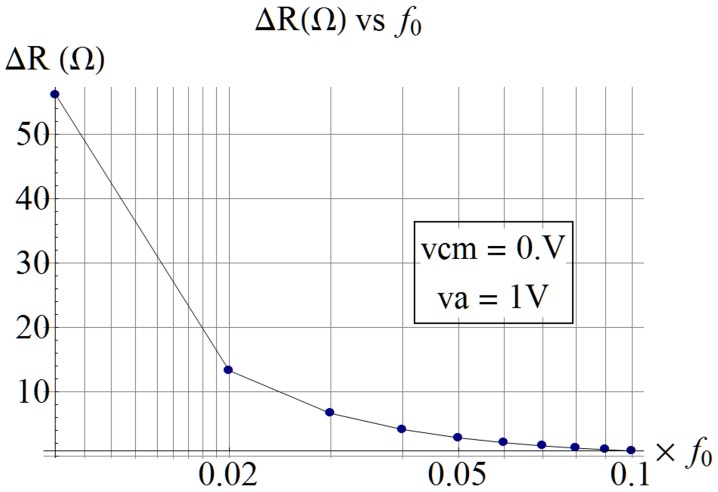
Frequency dependence of switching resistance. Resistance switching range decreases with increasing excitation frequency as observed by Radwan [18].

The preceding calculation confirms the expectation. When the device is completely filled with vacancies (if such an event is possible), the device is already at its highest resistance state compared to the pristine material. Substituting 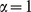 and calculating,
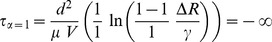



In absolute terms, it takes an infinite time to switch the device. The presence of the negative sign shows that from the perspective of the strict definition of transition time as the “time taken to transition from low-to-high resistance”, the device has already switched and is readily at its high resistance state. Thus it is expected that the possible range of switching resistance decreases with increasing concentration, which is the reason why the device has “already switched into high resistance” when 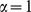. This intuition is confirmed by calculating and plotting the difference between the highest and lowest resistance, as a function of the normalized concentration 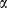. In Fig. 8, the memristor was exercised with a very low frequency sinusoid at one-thousandth the natural frequency 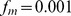, and having a common mode and amplitude of 0.25 V. The resistance range 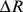 decreases at very high concentrations. Any benefit from increasing vacancy concentration is seen to top out for this particular example, at about *α = 0.2*. Fig. 9 demonstrates the reduction in the resistance range 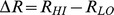 when the excitation frequency is increased as observed by Radwan et al. [18]. The same phenomenon is observable in general literature as I–V curves with a lobe size that is inversely proportional to the frequency of excitation [3]. Fig. 7(a) and Fig. 7(b) also demonstrate the same relationship between lobe size and excitation frequency.

Pickett et al. [19] address the topic of switching time from a constant current perspective. The result is that 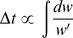 where 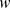 is equivalent to the accumulation boundary 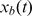. Their result is generated through a combination of “numerical integration motivated by physical insight from theoretical analyses, and trial and error modifications”. This paper by contrast is able to derive an analytical equation for transition time from the original PDE. It is expected that some “fitting” will be necessary to precisely match any experimental data. The equation (9) is tested against switching time from literature and the results are shown in Table 2, without normalization. The works of Nardi, Biolek and Strukov are for a vacancy mobility scenario like that assumed for this paper, while that of Liu is for a digital filament type memristor. The mobility for Liu's work was estimated from general reading and trial and error fitting into the switching time formula. The switching formula is seen to produce a reasonably good estimate for vacancy migration type of memristive devices and a satisfactory estimate for filamentary devices.

**Table 2:** Comparison of switching time 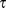 from literature to the prediction by the nonlinear transport model.

#	Author	Oxide	Transition	Transport Model			
			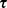		Unit	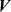	1Mobility
1a	[11] Nardi	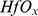	4	3.20	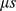	1.2	10^−08^
1b	-		15	20	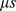	1.0	0.22×10^−8^
1c	-		20	21.7	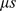	0.8	0.19×10^−8^
2	[12] Biolek	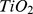	500	448	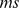	1.0	10^−14^
3	[03] Strukov	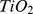	10	10	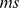	1.0	10^−14^
4	[13] Lu	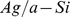	1.2	1.18	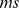	3.2	10^−08^

1 The mobility was estimated from among various general references. Rows 1b and 1c, use arbitrary mobility scaling to accommodate dependence on electric field.

In order to study the relationship between switching time and voltage, all the variables and terms in (9) except the programming voltage 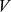 are assumed to evaluate to unity. Then it is possible to write 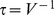. Taking the natural logarithm, 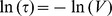 which is the same as 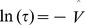 or 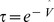. In essence (9) is equivalent to the statement in literature that switching time has an exponential dependence on the programming stimulus [14],[19]. Some papers choose to plot 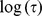 vs. 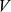 [13].

Menzel et al. [20] study temperature related effects on the switching transients. They produce the same primary equation (9) for switching time. Therefore (9) in this manuscript reveals the same influence of temperature on vacancy dynamics. Consider the secondary term 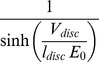 of Menzel's transition time. It can be re-written as 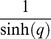 where 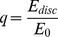 represents the field across the disc (concept from that paper) normalized with respect to the characteristic field 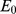. Assuming an average disc length, any increase in field strength will be due to a higher resistance that causes a larger voltage drop across the same disc length. For a disc field ranging from zero to infinity, the switching time ranges from infinity to zero. In other words, a zero resistance in the disc returns 
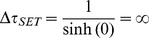
, where infinity means that there is no switching because the device is already at some immutable low-resistance. This corresponds to the case where (9) with 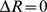 returns 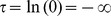, which in this manuscript means that the device is readily available in its high resistance state. When Menzel's device experiences a large or infinite disc resistance, their formula yields 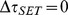 which again can be interpreted to mean that the device is already at its highest resistance, without any switching action. This manuscript with 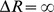 in (9) returns infinity which is taken to mean that the device will never switch. The seeming difference between the two quantities is redressed by proper interpretation, similar to interpreting the concept of infinity in root locus diagrams from classical control systems.

In summary, switching time exhibits an exponential relationship to voltage, and vacancy mobility. Switching time has quadratic dependence on the device length making device length a dominant factor that influences operating speed. Increasing the vacancy concentration will result in a decrease in the switching resistance range, negating the reduction of switching time. Increasing the frequency of excitation also decreases the switching resistance range.

### F. Standard Memristive Equation

The standard memristive equations attributed to Chua and as presented in [3] are,
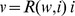


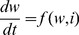
(10)


Where


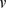
 is the stimulus voltage,


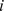
 is a current that results in a charge passing through the device,


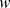
 is a state variable equivalent to the 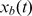 or the normalized 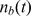 in this paper.


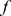
 could be a function of time,


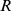
 is a resistance and could be a function of time.

The first condition can be re-stated as 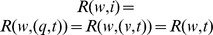. This subsection will test the proposed quasi-linear model for adherence to the standard memristive form.

Starting from (7) with 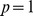 for convenience,
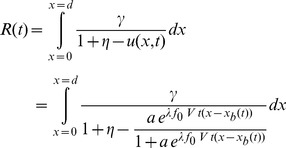
(11)


Thus resistance is a function of 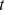 and 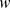 (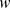 is represented equivalently by 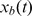 or the normalized 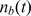 in this paper), satisfying the first condition 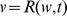.

Starting from the normalized version of (5) and differentiating with respect to time results in the following equation, shown with 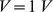 and 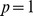.
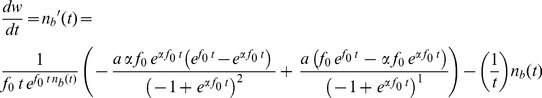
(12)


The above equation fits the expectation that 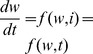, where this paper substitutes 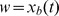 or the normalized 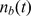.

In summary the model presented here fits the profile for a general memristive system as shown by (11) and (12).

### G. Mathematical Classification

This paper has so far demonstrated that the model (1) and its particular solution (2) are able to generate a variety of information about the memristor, comparable to independently published experimental and theoretical data. This subsection will investigate a mathematical classification for the model, based on order, linearity and homogeneity.

Equation (1) is a form of the continuity or transport equation with a variable coefficient. It is also found referred to in literature as a variable coefficient advection equation [15]. A general PDE has the form 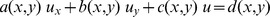. The case presented here is equivalent to the simpler 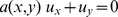, with 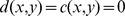, 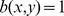 and 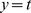 [21].

#### 1. Order

The equation 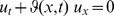 contains only derivatives to the first order, making it a first-order equation.

#### 2. Homogeneity

Every term in the given PDE contains either *u(x,t)* or some derivative of 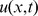 . Specifically there are no terms with any other functions other than some form of the solution function 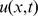 in it. Therefore the equation is homogeneous.

#### 3. Linearity

First order PDEs may be classified as linear or nonlinear. There are various degrees of nonlinearity such as semi-linear, quasi-linear or fully nonlinear. A PDE is semi-linear when the coefficient of the highest derivative of *u* does not depend on 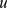. A PDE is quasi-linear when the coefficients of the highest order terms may depend on 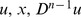 but never 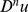, where 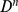 represents the 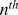 derivative. A PDE is fully nonlinear when the highest order derivatives appear non-linearly in the equation, such as 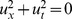. This example equation can be written as 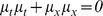 by pulling out one of the derivatives as a coefficient to show that it is fully nonlinear because of the presence of derivatives of the kind 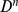 as coefficients.

Consider the simple case where programming voltage and mobility are constants in the model under consideration. Then it is possible to write the coefficient 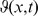 as 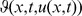.
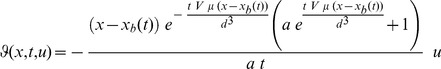



At the other extreme, where the programming voltage is a function of time and mobility is a function of the spatial location inside the device, it is still possible to write the coefficient 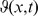 as 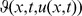.
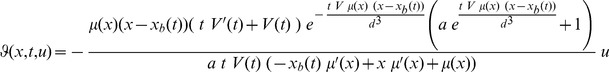



In the two preceding equations 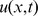 was abbreviated as 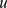. Thus, it is possible to express the coefficient 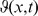 as 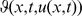, when the accumulation boundary can be expressed as a function of an average 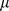, allowing straightforward computation of 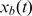. The coefficient of the highest derivative is shown to depend on *u(x,t)* therefore the model is quasi-linear.

In summary this subsection shows that the governing equation presented in (1) may be classified as a first order, homogeneous, quasi-linear PDE.

### H. Circuit Implementation

This subsection looks at a simple passive low-pass filter circuit implementation using a memristor. A circuit topology is presented and the intuitive outcome is verified by simulating the proposed electrical network mathematically, using the model proposed in this paper.

#### 1. The Memristor-Capacitor Filter

**Figure 10:**
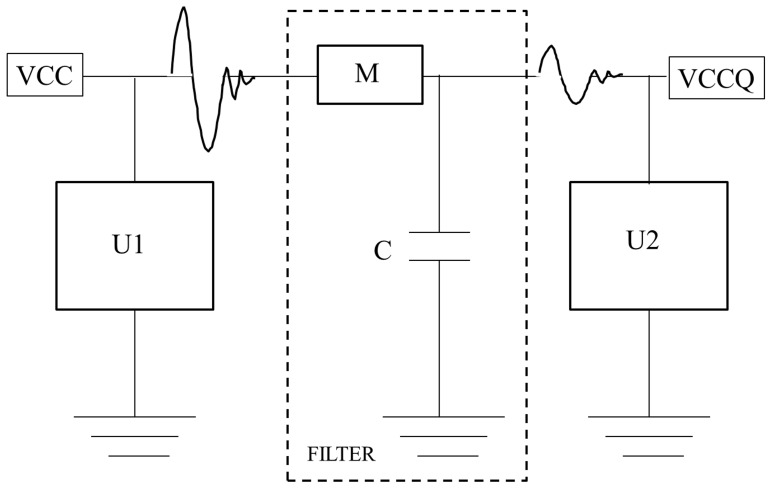
Sketch of Memristor-Capacitor (M-C) filter implementation. Circuit U1 can be a digital circuit that generates and couples noise into the power grid VCC. The circuit U2 may be an analog circuit that expects a quiet supply VCCQ. The memristor-capacitor (M-C) circuit serves to isolate the two.

The test circuit consists of a memristor-capacitor (M-C) combination constructed similar to a textbook resistor-capacitor (R-C) filter. Consider an application where a M-C filter might be used to isolate the power supply of a circuit, from a more noisy side of the same supply. The circuit topology is shown in Fig. 10. Circuit U1 is some digital circuit that generates noise into the power grid and isolated from circuit U2 by the M-C filter in the dotted box. The power supply net named VCC is being yanked around by U1. The side labeled VCCQ is the quiet supply and expects to see only a filtered version of the noise.

The simulation compares the result of using an M-C filter versus a regular R-C filter, where the resistance R is set to the lower resistance value of the memristor, namely 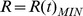. The low R guarantees a startup transient for the R-C, comparable to the M-C circuit. It is assumed here that the memristor has been programmed by some means into its low-resistance state prior to usage in the circuit.

The 3 dB bandwidth of the filter will be inversely proportional to the memristance. Therefore, upon initial application of power, the R-C and M-C circuits should exhibit similar power-up transients. The memristor however will soon transition into its high-resistance state causing the M-C filter to have a lower bandwidth than the R-C version. The lower bandwidth is expected to translate into better noise rejection on the VCCQ net.

#### 2. Simulation

**Figure 11:**
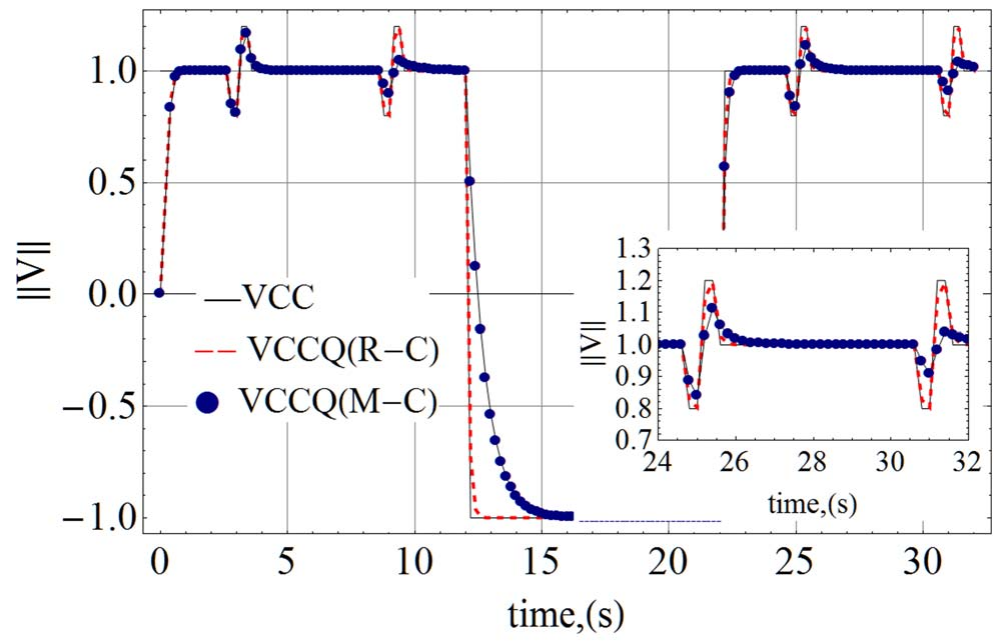
Simulated result comparing the M-C and R-C filters. The M-C filter was initialized into its low resistance state and has the same power-on transient performance as the R-C filter. After power on, the M-C filter is able to attenuate noise better than the R-C filter as visible in the inset.

The simulation result is shown in Fig. 11. The input power supply at the net VCC is toggled 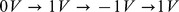. The initial 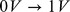 is a power on event. The next 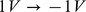 transition is a power off event including a negative excursion to reset the memristor. The final pair 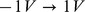 is a second power on event. The input wave is the solid black thin line. The output at VCCQ due to the R-C filter is the dotted red line, and the result of M-C smoothing is the thin line with filled navy blue circles. The first and second power on edge is similar for the R-C and M-C filters. There are two noise events of ±200 mV at one-third and two-thirds of the time into each power-on event. The M-C filter does a much better job of attenuating the second noise event, because the memristor has transitioned into a higher resistance, thereby reducing the bandwidth of the filter. The inset shows a zoomed in view of the noise and attenuated outputs during the second power-on event. The dotted line for the R-C filter more or less (undesirably) follows the input noise while the memristor attenuates each subsequent noise pulse with (desirably) increasing attenuation.

An important consideration is that while the transient is comparable during the power-on rising edge, the M-C is slower on the falling edge during power off. This is because the memristor is at a higher resistance, and only starts to decrease in resistance as the power supply collapses. The intention with two power-on cycles is to show that when considering an ideal memristor, the behavior is repeatable. If an application desires the rapid on/off performance, the transition from high to low resistance might be a minor performance limiter.

Nonetheless this subsection has demonstrated a simple application where a memristor may be used to implement a variable bandwidth low-pass filter that resets automatically. The automatic reset assumes that the device is not subject to performance variations, and transitions identically between low and high resistance. This assumption, while simplistic, is reasonable when considering the simple objective. This subsection also demonstrates that the derived model (8) is usable in a circuit network containing other electrical components, when biased properly.

## Discussion

This paper presents a computable nonlinear vacancy transport model for the memristor. Initially a word-problem is stated, based on the behavior of vacancies as described by Strukov and Williams [3], [4]. A governing equation (1) is stated based on the general consensus that a non-linear drift [6], [14] is responsible for vacancy migration, under the action of a programming voltage. The proposed solution (2) uses a variable velocity term 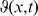 that will accommodate the nonlinear drift. The solution is successfully subjected to mathematical verification.

The present work shows that vacancy velocity can be calculated as an analytical equation (6). Fig. 2 shows the expected nonlinear vacancy velocity toward the device ends. In addition to vacancy velocity, (4) is also able to track the transit of the accumulation boundary as evidenced by the change in the sign of the calculated velocity. Equation (5) locates the accumulation boundary at any time, while (6) quantifies the newly introduced concept of vacancy symmetry boundary. The vacancy symmetry boundary is different from the accumulation boundary in that it always locates itself inside the device boundary. The normalized accumulation and vacancy symmetry boundary are shown in Fig. 3. The present work derives the formula for device resistance. Fig. 4 plots the resistance-time transient showing agreement with a plot generated using the formula from Joglekar and Wolf [5]. Equation (8) is a computable formula for the two variable resistors that match the dual variable resistor model from Strukov and Williams [3]. Complex plane plots are presented in Fig. 5, showing the phase dependence for the sub-resistors 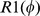 and 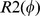 and demonstrating that the total resistance 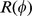 is a real number. The significance of (8) and Fig. 5 taken together is that they confirm the observations about negative resistance made by Chua [16]. The transition time formula (9) has the main term in full agreement with [14], [17], [20], while the enhancement term suggests within reason that switching time should also be a function of the vacancy concentration. With proper interpretation of the results, the enhancement term exhibits the same behavior as in [20]. A subtle inverse relationship between switching resistance range and vacancy concentration is computable and shown in Fig. 8. Frequency dependence of the switching resistance range 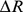 as read from [18] and indirectly observable in the reduced lobe-size of high-frequency I–V curves in literature is confirmed by Fig. 9, where the data was generated using (8). Equations (11) and (12) show that the model has the general form expected by Chua's theoretical formulation. The author classifies the model as a first order, homogeneous, quasi-linear PDE.

A simple low-pass filter implementation is presented to demonstrate that the resistance formula (8) is usable in a circuit design context. The simulation result in Fig. 11 shows that the memristor is able to function as a self-resetting, variable bandwidth low-pass-filter as expected from its construction.

All derivations, formula manipulations and symbolic computations were done using Mathematica 7 [22] on a Windows 7 PC with a 2.5 GHz Intel Core i5 CPU. The runtime for each derivation, validation or plot generation program was less than five minutes.

## Conclusions

Through independent methods, this paper presents a governing PDE and analytical solution by which the contemporary high level dual variable resistor abstraction of the memristor from HP's Strukov and Williams is shown related to vacancy dynamics. The solution satisfies the governing PDE. Through algebraic manipulations, the model yields the same or enhanced versions of results and observations already known in general literature. The enhancements are tested against intuition, explained and where possible compared to independent experimental data. New insights such as vacancy velocity, vacancy symmetry boundary and the dependence of transition time on vacancy concentration are presented with supporting derivations. The constituent resistors of the dual variable resistor model are quantified with equations and the reason for the existence of a negative resistance region in the I–V curves is demonstrated mathematically. The model is shown to be viable for circuit analysis by using it in a simple M-C filter. All the variables used in this modeling effort are defined and the equations presented are unambiguously computable. The values used for the various plots are tabulated in Table. 1 and were obtained from the various references or assigned a reasonable value when not available from literature. Table 2 shows absolute transition time predictions made using (9) and compared to reference literature. Table 3 summarizes the memristive phenomena that are reproduced by this model. This manuscript derives all memristive characteristics from (1) and (2), while the external references in Table 3 develop them in a piecemeal fashion.

**Table 3:** Table of memristive characteristics reproduced by the nonlinear transport model.

#	Memristor characteristic	External References	This manuscript, Fig./(Eqn.)
1	Governing equation	-	(1)
2	Particular solution	-	(2)
3	Current-Voltage curves	1, 2, 3, 4	Fig. 6, Fig. 7
4	Frequency dependence of lobe size	3, 4	Fig. 7
5	Dual variable resistance	3	Fig. 4, Fig. 5, (8)
6	Negative Resistance	16	Fig. 6, (8)
7	Non-linear evolution of boundary	5, 9	Fig. 3, (5)
8	Non-linear evolution of vacancy concentration	8	(2)
9	Nonlinear vacancy velocity	6, 14	Fig. 2, (4)
10	Transition time	14, 17,21	Fig. 4, (9)

The author recognizes that no single equation can claim to model any phenomenon exactly. Models benefit from tuning knobs that accommodate various non-idealities. The proposed quasi-linear governing equation is meant to demonstrate a unifying model that exhibits observed memristive characteristics reasonably well. This manuscript hopes to have achieved that objective with the many supporting equations, plots and comparative analysis with respect to independent empirical and theoretical literature.
